# Geographical information system (GIS) as a new tool to evaluate epidemiology based on spatial analysis and clinical outcomes in acromegaly

**DOI:** 10.1007/s11102-013-0548-3

**Published:** 2013-12-25

**Authors:** Luciana Ansaneli Naves, Lara Benigno Porto, João Willy Corrêa Rosa, Luiz Augusto Casulari, José Wilson Corrêa Rosa

**Affiliations:** 1 Unit of Endocrinology, Faculty of Medicine, University of Brasilia, Brasília, Brazil; 2 Institute of Geosciences, University of Brasilia, Brasília, Brazil

**Keywords:** Acromegaly, Pituitary tumor registry, Geographical information system

## Abstract

Geographical information systems (GIS) have emerged as a group of innovative software components useful for projects in epidemiology and planning in Health Care System. This is an original study to investigate environmental and geographical influences on epidemiology of acromegaly in Brazil. We aimed to validate a method to link an acromegaly registry with a GIS mapping program, to describe the spatial distribution of patients, to identify disease clusters and to evaluate if the access to Health Care could influence the outcome of the disease. Clinical data from 112 consecutive patients were collected and home addresses were plotted in the GIS software for spatial analysis. The buffer spatial distribution of patients living in Brasilia showed that 38.1 % lived from 0.33 to 8.66 km, 17.7 % from 8.67 to 18.06 km, 22.2 % from 18.07 to 25.67 km and 22 % from 25.68 to 36.70 km distant to the Reference Medical Center (RMC), and no unexpected clusters were identified. Migration of 26 patients from 11 others cities in different regions of the country was observed. Most of patients (64 %) with adenomas bigger than 25 mm lived more than 20 km away from RMC, but no significant correlation between the distance from patient’s home to the RMC and tumor diameter (r = 0.45 *p* = 0.20) nor for delay in diagnosis (r = 0.43 *p* = 0.30) was found. The geographical distribution of diagnosed cases did not impact in the latency of diagnosis or tumor size but the recognition of significant migration denotes that improvements in the medical assistance network are needed.

## Introduction

The development of pituitary adenomas may be related to several pathophysiological factors that have been described in recent years. Although the exactly causative mechanisms involved remain elusive, they might include genetic and epigenetic aspects, resulting in cell cycle disruption, cell signaling abnormalities, oncogene activation, loss of tumor suppressor activity, and receptor and hormone signaling dysfunction [1–5].

Despite the advances in comprehension of mechanisms involved in the development and morbidity of somatotropinomas [6–15, the influence of environmental and socioeconomic factors in the outcome of acromegaly has not yet been properly evaluated [16]. The recognition of regional aspects and geographical distribution of diagnosed cases may help to identify probable clinical case location, and to describe epidemiological data.

The frequency of pituitary tumors is not well established, and most of them are under diagnosed. A metanalysis suggested a frequency in general population in the USA, of 14.4 % e 22.5 % in autopsy and radiological series respectively [17] and prevalence of 190–280 cases/million [18]. An european study have shown a presence of clinically relevant pituitary adenomas in 1:1,064 inhabitants [19]. The prevalence of acromegaly in different multicentric and international registries is around 60 cases per million [20–22]. In Brazil there is no published national database registry for the disease, but in the study region, the population is around 2,562,963 inhabitants, the expected number of cases should be around 150 patients diagnosed in Brasilia. We describe a representative cohort of 112 patients, in regular medical care in our medical center.

In recent years, geographical information systems (GIS) have emerged as a group of innovative and important software components of many projects in public health and epidemiology [23]. The usage of GIS tolls in health sciences provides scientific and accurate ways to register, analyze, and map disease information. GIS tools include processing geographic data, topographic databases, satellite remote sensing imagery, among other databases, to create maps and to cross-correlate public health registries and other clinical records to environmental factors. This data analysis scheme may lead into the investigation of the relation of clinical aspects to the mentioned geographical parameters, and to describe disease variables and inter-relationships based on its observed spatial distribution [24, 25]. It may also be useful in disease surveillance based in the identification of possible spatial clusters of disease occurrence [26] and to aid planning strategies to improve regional health care [27–29].

Until now, the use of GIS has not been applied to pituitary tumors, hence the methodology is novel and the study represents a proof of principle with this mapping technique, to evaluate geographical pattern of acromegaly distribution in Brazil.

### Regional and socioeconomic aspects

Brasilia is located around the middle of the polygon that defines the Federal District of Brazil, at an altitude more than 1,000 m over the sea level. The city was constructed in 1960, and planned to be similar to an airplane shape, with thirty others administrative regions, called satellite cities, located around the center and in the vicinities of the limits of the district. Most of the population lives in the western portion of the Federal District and there is a poorly populated rural area in the eastern part of the region. In this region, geochemical anomalies and occurrence of ores with radioactive components were not identified, comparing to the rest of Brazilian soil [30, 31]. The Federal District is not a region rich in industrial or environmental contaminants. A recent study conducted by the University of Brasilia evaluated the concentration of chemical elements in an important watershed of the Federal District. It was shown the absence of manganese and copper in the water, excluding the possibility of water contamination from industrial activities in the study area [32].

According to Brazilian Census 2010 [30], the population of the Brasilia area represents a completely heterogeneous sample of an almost unbiased population, composed by several ethnic origins and people that have migrated from several different regions of the country, to built the planned city, that was recently constructed in a previously inhabited rural area of the country. The economics conditions of the main city (Brasilia) are privileged, but considering the Federal District as whole, the population is basically composed of middle- and working-class Brazilians [30]. The local public health system is composed by a network of health posts for primary health care, hospitals dedicated to secondary care, and two hospitals for tertiary care. Most of patients with diagnosis of neuroendocrine disorders are referred to our center. The spatial analysis was applied in order to investigate possible trends in Acromegaly in a representative cohort of the Brazilian population, using a tool that was firstly applied to pituitary tumours.

## Objectives

The aims of this study were (1) to demonstrate and validate a method to link acromegaly registry data with a GIS mapping and analysis program; (2) to describe the spatial distribution of acromegaly in the study region in order to identify the presence of disease clusters; and (3) to analyze the difficulties of access to Health Care System and its impact in latency for diagnosis and tumor size.

## Methods

### Referral medical center (RMC)

The University Hospital of Brasilia is a RMC and belongs to the tertiary care network of the Federal District. The Neuroendocrine Unit is a referral center for treatment of pituitary diseases in the central region of Brazil and maintains a registry of all patients with pituitary tumors diagnosed since 1995. All clinical information is updated periodically using electronic medical records.

### Geographical region of interest

Brasilia is a planned city, located in the center-western region of Brazil, constructed in 1960 to be the national capital. It is part of the Federal District of Brazil, which population is comprised of people that have migrated from several remote regions of the country in the past 50 years. So, demographic studies show that the current population of the Federal District represents a sample of a completely, and maybe unique, multiracial group. Information on the socioeconomic conditions concerning this region were provided by the Brazilian Institute of Geography and Statistics (IBGE) and the data collected from the most recent Brazilian Census, 2010 [30, 31].

### Acromegaly registry data

Patient data were obtained by examining the Neuroendocrine Unit electronic medical records using a proper commercially available software tool (Access, Microsoft, Bellevue, WA), and a locally developed computer program, designed to collect significant information related to pituitary function and disease. All patients with diagnosis of acromegaly who had a clinic visit, surgery or radiotherapy on this unit, in the time range from 1995 to 2013, were included. Patient variables considered were address, age, sex, signs and symptoms at diagnosis, comorbidities, as well as all other data concerning laboratorial and radiological findings. The study was performed in accordance with the declaration of Helsinki, and was approved by the local ethics committee.

### Application of acromegaly registry data to GIS mapping tool

The Brasilia acromegaly registry data was incorporated into a spreadsheet usable format (Excel 2003, Microsoft, Bellevue, WA). These data were converted to a database file structure for input to the chosen GIS software (namely, ArcGIS version 9.3, ESRI, Redlands, CA). The residential addresses reported by participants in the past 10 years prior to diagnosis were selected for the spatial analysis. The address information for cases in which residency time in the Federal District began after the diagnosis were excluded. Geocoding parameterization was used in order to obtain coordinates of the home address of each patient. To ensure protection of the privacy of individuals, as recommended from Health Insurance Portability and Accountability Act (HIPAA) and IOM privacy regulations [33–35], and to avoid misrepresentation in our data, the chosen scale of the maps was 1:300,000 and no geocodes (latitude and longitude) were shown. This way, each point representing the residence of an individual, includes a larger area, including at least 15,000 people. The maps were then generated placing residences in a non-disclosed grid, using a proper map scale that would not display streets or individual residences, and preserve individual confidentiality.

## Statistics

All results were expressed in mean or median, the individual groups of patients were compared and, whenever applicable, the Pearson correlation coefficient was calculated. Student’s t test was performed for the comparative analysis of quantitative variables. The Fisher exact test was used to compare frequencies and determine associations between categorical variables. All statistical analyses were conducted using the Statistical Package for Social Sciences (SPSS 17.0) and *p* values < 0.05 were considered statistically significant.

## Results

### Cohort

The cohort was composed of 112 patients, diagnosed in our center from January 1995 to January 2013. The gender distribution was 47.3 % women, and 52.7 % men. The mean age at diagnosis was 42.1 years (ranging from 14 to 70), and 60.4 % of patients had their age ranging between 31 and 60 years old at the first endocrine evaluation. The most frequent symptoms and co-morbidities are described on Table 1 compared to tumor size and invasiveness.

**Table 1:** Symptoms at diagnosis in patients with acromegaly categorized by tumor size and invasiveness before treatment

Symptoms at diagnosis	Size of pituitary tumors		Invasive	*p*
	Microadenomas	Macroadenomas		
Symptoms				
Headaches	7	31	16	0.01*
Visual impairement	6	18	9	0.02*
Sweatness	8	17	9	0.40
Soft tissue	78	97	90	0.60
Artralgias	9	22	13	0.02*
Galactorrhea	1	8	2	0.25
Hypogonadism	5	17	5	0.10
Co-morbidities				
Diabetes mellitus	3	9	8	0.20
Hypertension	6	17	10	0.10

The *p* values, were calculated by analysis of variance (ANOVA)

* The *p* values were considered statistically significant if < 0.05

### Delay for diagnosis

**Fig. 1:**
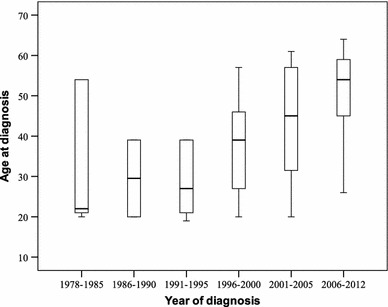
Relation between age distribution at first medical evaluation and year of diagnosis

The mean delay time between first symptoms and diagnosis was 5.8 years (original data ranging from 4 to 9 years), and the median was 5 years. The older patients presented longer duration of symptoms preceding diagnosis (*p* = 0.003 *r* = 0.73). For patients who referred to live outside the Federal District by the time of diagnosis, the mean delay time was 6.8 years, and the computed data median was 7 years. In most patients, the disease was identified in the last 10 years, and for 29.2 % of the patients, the disease was diagnosed from 2007 to 2013. Despite better tools for precocious diagnosis, we found that the age pattern at the diagnosis did not improve along time (Fig. 1).

### Application of geographical information system

#### Spatial analysis of cohort of patients

**Fig. 2:**
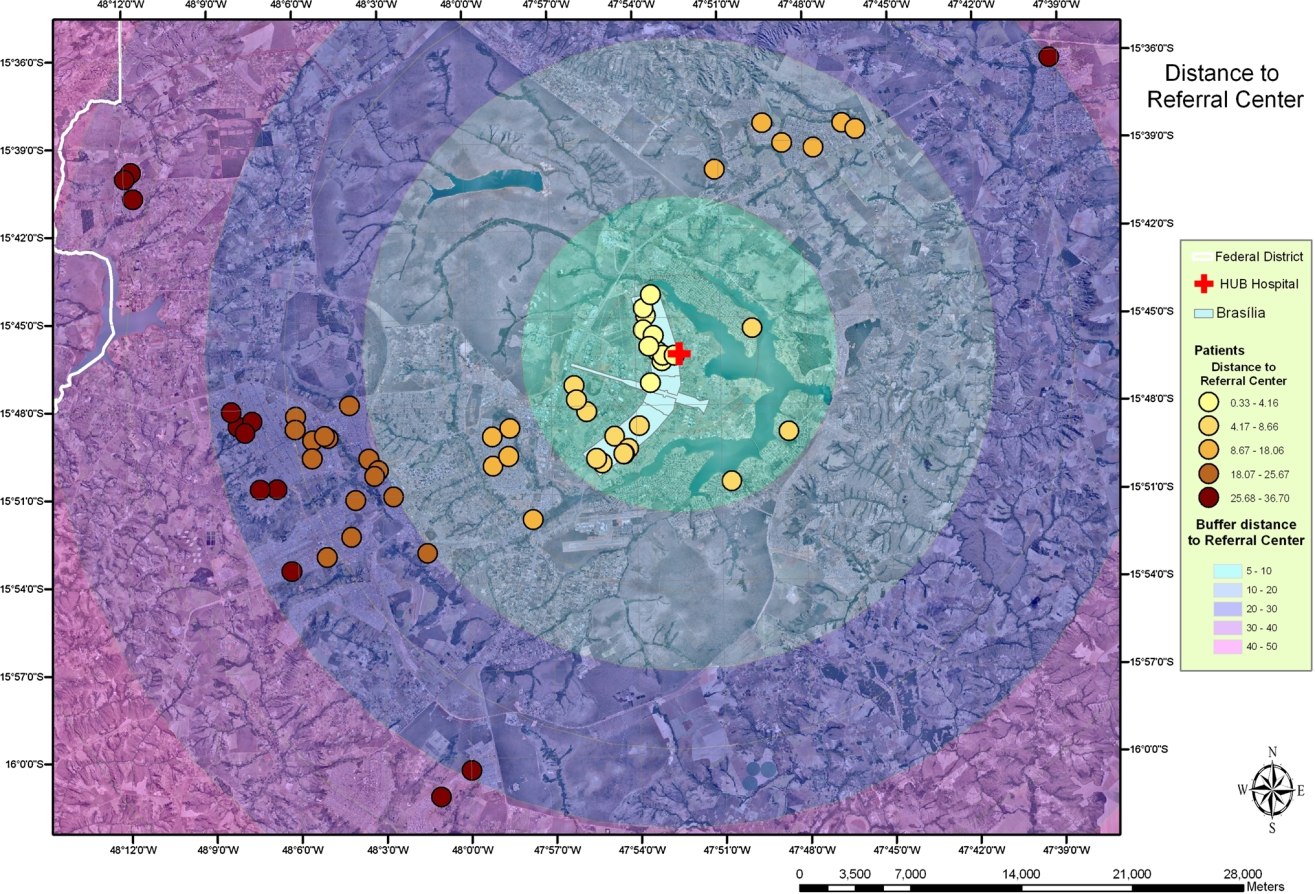
Patients’ home distance to referral clinical center. *Buffer distances* are represented in five zones from 5 to more than 50 km long, and patients’ clusters in *different colors* according to region. GIS software (ArcGIS version 9.3, ESRI, Redlands, CA).* Map scale* 1:300,000

The Federal District was represented in buffers corresponding to the radial distance to medical care unit. Five zones were segmented each 10 km away from the RMC. The analysis of buffer distribution showed that 38.1 % of patients lived from 0.33 to 8.66 km, 17.7 % from 8.67 to 18.06 km, 22.2 % from 18.07 to 25.67 km, and 22 % from 25.7 to 36.7 km distant to the RMC (Fig. 2). For this analysis we considered patients living in the Federal District 10 years prior to diagnosis. No unexpected clusters were found. The highest concentrations of patients corresponded to areas with higher population density.

**Fig. 3:**
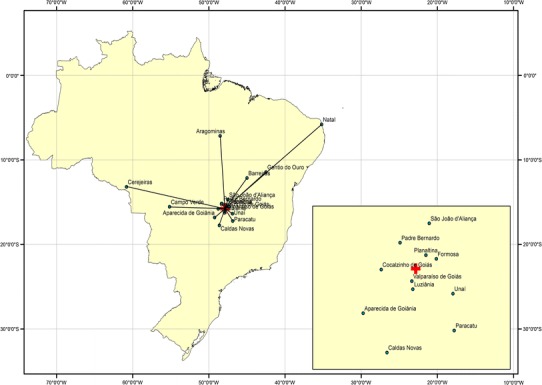
Map of pattern of migration of patients from other regions in Brazil, that looked for assistance at Neuroendocrine Unit of the University Hospital of Brasilia

For the spatial analysis, from the 112 initial records, 24 % were left unmatched, related to incorrect information of the address location. Most of these errors were identified to be due to abbreviated street names, inadequate house numbers, and county location. Some unmatched records corresponded to patients living in distant regions of the country, but referred a local address to benefit from the health services in Brasilia. Those addresses were reviewed and correctly matched to the residence addresses by the time of the diagnosis. Twenty-six patients had a fixed residency, 10 years prior to diagnosis, located in 11 cities from different regions of the country, and have migrated to Brasilia to look for attendance at the Neuroendocrine Unit of the University Hospital of Brasilia (Fig. 3).

#### Tumor volume and distance to the referral medical center

The frequency of microadenomas was not different among the two genders (*p* = 0.80). Women presented macroadenomas more frequently (*p* = 0.03), and invasive lesions were more common in men (*p* = 0.01). Microadenomas corresponded to 22.3 % of cases and 43.8 % of patients had invasive lesions by diagnosis.

**Fig. 4:**
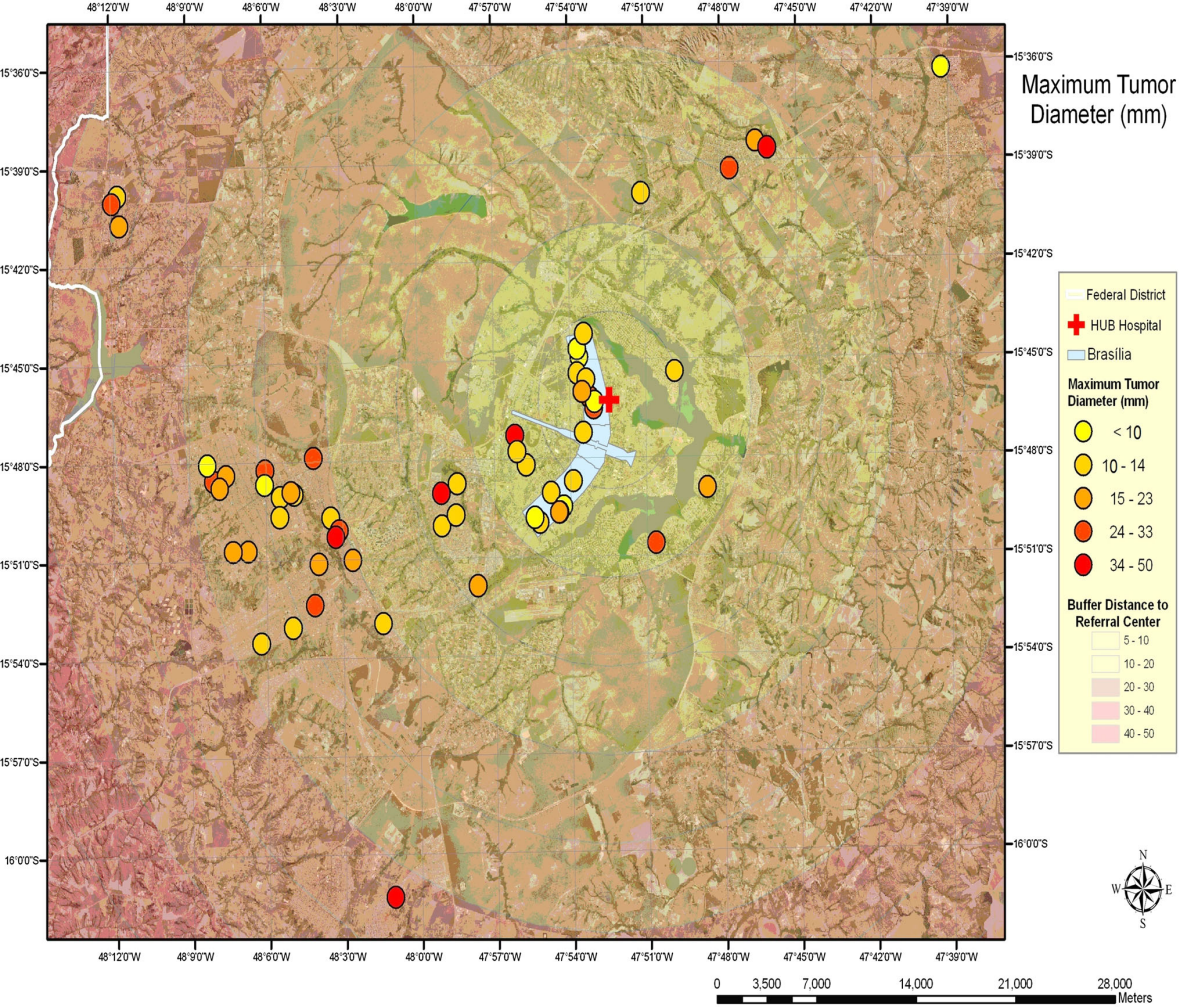
Spatial distribution of patients according to the maximal tumor diameter and distance to reffered medical center. GIS software (ArcGIS version 9.3, ESRI, Redlands, CA). *Map scale* 1:300,000

The patients were represented by different colors and plotted in a map, according to their tumor larger diameter at diagnosis. The map was divided in 5 buffer zones ranging from 5 km to more than 50 km. Sixty-four percent of patients with macroadenomas bigger than 25 mm of maximum tumor diameter lived more than 20 km away from the RMC (Fig. 4). There was no significant correlation between tumor size and distance to the hospital (*r* = 0.45 *p* = 0.20).

There was no significant correlation between the latency for diagnosis and the distance from the patient′s home address to the RMC (*r* = 0.43 *p* = 0.3), even considering the patients living out of the Federal District.

## Discussion

In our survey, we verified that the most invasive lesions were found in younger patients, and in cases of FIPA, corresponding to previous studies which found a higher potential of aggressiveness in these patients [4, 36]. The delay time between the onset of symptoms, and the recognition of the disease was shorter than usually described [37, 38]. It is possible that once the disease was suspected, the patients had easily been routed to our institution, which is a referral to neuroendocrine pathologies, and this may have reduced the time for definitive diagnosis. It is also remarkable that the diagnosis of acromegaly has risen in the last years in our center, which may reflect the concern of the local general practitioner physicians, and regional endocrinologists, to identify and refer these patients to our center. An improved local knowledge may be related to a larger number of talks of endocrinologists from our group in other local institutions, as well as the result of a better-structured endocrinology syllabus in the main medical school of Brasilia, which is related to our center.

Despite the diagnostic tools and medical information about the disease, the age by diagnosis did not reduce along decades. Curiously, we observed that in 80’s and early 90’s the mean age at diagnosis was younger than in recent years, probably because at that time, only the patients with more aggressive tumors reached the medical health care in our city.

Regarding to GIS mapping, this is the first study known to us which uses this method to evaluate the epidemiology of acromegaly. So, we are still not aware of all possible applications of our results. It is possible that environmental features may influence the appearance and evolution of the disease. Cannavò et al. [16] showed a higher prevalence of this infirmity in a highly polluted area in Italy. The Federal District of Brazil is an administrative area, with low concentration of dangerous environmental hazardous sources. The vast majority of patients live in urban neighborhood, which includes mostly small apartment buildings and houses, and none of them worked previously in industries or large farms. So, the population described in this study was homogeneously urban, with comparable socio-cultural conditions and reasonable access to medical system. All georreferenced areas considered the patient’s addresses in the past 10 years prior to diagnosis, including those located out of the Federal District. In this study, we did not identify environmental factors that could influence the occurrence of acromegaly. GIS tools offer a visual way to identify clusters, and in our survey no unexpected clusters were found.

In oncology, GIS has been used with promising results. Spatial epidemiology using individual level data from population-based studies may bring forth new exposure hypothesis. GIS analysis of cancer may reveal inducing factors and give clues for possible prevention measures [39, 40]. Although we found no differences related to the spatial distribution of acromegaly in the Federal District, variations may emerge while applying the GIS techniques to a larger cohort of patients. GIS mapping is also a useful method for planning health care assistance. Patients from different states looked for attendance in our center, suggesting that delivery of Brazilian health care may improve with proper location of new health centers in the most needed areas. GIS mapping may also be an important tool while planning future centers for medication distribution in areas that lack proper public health care.

## Conclusion

GIS is a reliable method to evaluate the mapping of acromegaly in different ways related to the spatial distribution of the disease. In the future, it may provide guidance for comprehension of environmental features that might affect the pathogenesis of the pituitary tumors. GIS can also help Health Systems in their mission to improve assistance. Our work shows an example of a useful application of GIS analysis applied to data sets now available in electronic medical records. Even though we could not pinpoint a definite link between the occurrence of acromegaly and the local environmental problems, we showed that, with the properly organized medical records, it is possible to identify health diagnosis patterns and propose local improvements in the medical assistance network. It is the first to evaluate if the access to health care could impact the outcome of the disease, using a mapping technic.
